# Classifying patients with invasive fungal disease: towards a unified case definition?

**DOI:** 10.1093/jac/dkaf261

**Published:** 2025-07-24

**Authors:** Mehmet Ergün, Roger J M Brüggemann, Alexandre Alanio, Robbert G Bentvelsen, Karin van Dijk, Meltem Ergün, Katrien Lagrou, Jeroen A Schouten, Joost Wauters, P Lewis White, Paul E Verweij

**Affiliations:** Radboudumc-CWZ Center of Expertise for Mycology, Radboud University Medical Center, Nijmegen, The Netherlands; Department of Medical Microbiology, Radboud University Medical Center, Nijmegen, The Netherlands; Radboudumc-CWZ Center of Expertise for Mycology, Radboud University Medical Center, Nijmegen, The Netherlands; Department of Pharmacy, Radboud University Medical Center, Nijmegen, The Netherlands; Mycology-Parasitology Department, Hôpital Saint-Louis, Assistance Publique Hôpitaux de Paris, Paris, France; National Reference Center for Invasive Mycoses and Antifungals, Translational Mycology Research Group, Mycology Department, Institut Pasteur, Université Paris Cité, Paris F-75015, France; Microvida Laboratory for Microbiology, Amphia Hospital, Breda, The Netherlands; Department of Medical Microbiology, Leiden University Medical Center, Leiden, The Netherlands; Department of Medical Microbiology and Infection Control, Amsterdam Infection & Immunity Institute, Amsterdam UMC, Vrije Universiteit Amsterdam, Amsterdam, The Netherlands; Faculty of Mathematics and Computer Science, Eindhoven University of Technology, Eindhoven, The Netherlands; Department of Microbiology, Immunology and Transplantation, KU Leuven, Leuven, Belgium; Department of Laboratory Medicine and National Reference Center for Mycosis, University Hospitals Leuven, Leuven, Belgium; Department of Intensive Care Medicine, Radboud University Medical Center, Nijmegen, The Netherlands; Scientific Centre for Quality of Healthcare (IQ Healthcare), Radboud Institute for Health Sciences, Radboud University Medical Center, Nijmegen, The Netherlands; Department of General Internal Medicine, Medical Intensive Care Unit, University Hospitals Leuven, Leuven, Belgium; Public Health Wales Mycology Reference Laboratory and Cardiff University Centre for Trials Research, University Hospital of Wales, Heath Park, Cardiff, UK; Radboudumc-CWZ Center of Expertise for Mycology, Radboud University Medical Center, Nijmegen, The Netherlands; Department of Medical Microbiology, Radboud University Medical Center, Nijmegen, The Netherlands

## Abstract

Management of invasive fungal disease (IFD) is increasingly challenging due to recognition of novel at-risk groups, emergence of new fungal pathogens and antifungal drug resistance. Together with the availability of new diagnostic tests and treatment modalities, robust and broadly applicable IFD case definitions are critical to support research. However, the ability to classify IFDs with the current definitions has decreased, prompting the development of new case definitions. Furthermore, current case definitions rely on a single positive test as mycological evidence, while not considering discordant evidence. We propose to explore the development of a machine learning (ML)-based IFD classification model, which uses algorithms to automatically ‘learn’ from observed data to consistently and accurately classify IFDs. Although developing and validating an ML-based IFD classification model is a significant undertaking, such an endeavour should be considered a worthwhile investment by the mycology community to standardize and reduce the ambiguity in the diagnosis of non-proven IFD.

## Introduction

The landscape of invasive fungal diseases (IFDs) is dynamic, with emerging fungal pathogens (e.g. *Candida auris*), developing antifungal drug resistance and newly recognized host groups, including those with severe respiratory viral infections (i.e. influenza and severe acute respiratory syndrome coronavirus 2 infection).^1,2^ Rapid commercial diagnostic tests are being increasingly used to diagnose IFD, and genomic sequencing tools increase our abilities to identify and track migration of resistance and support infection control interventions. Finally, new antifungal drugs and host-directed therapies are under development that will broaden our therapeutic arsenal and potentially improve the outcomes of patients with difficult-to-treat IFDs.^3^

The increased complexity of IFDs, including heterogeneous patient risk profiles, intrinsic and acquired drug resistance, drug interactions and toxicity, challenges established treatment strategies both empirical and pre-emptive. Research is needed to support evidence-based interventions focusing on One Health aspects, fungal disease surveillance, multi-component diagnostic test validation and clinical trials. For each of these standardized IFDs, case definitions are critical to enable international comparisons and minimize disparities. Standardization of definitions of IFDs for clinical research was the main reason for developing the European Organization for Research and Treatment of Cancer (EORTC) and the Mycosis Study Group Education and Research Consortium (MSGERC) consensus definitions.^4^ These definitions incorporate host factors, clinical factors and mycological evidence. Although originally the scope was restricted to patients with cancer and recipients of haematopoietic stem cell transplants, other patient groups were included in the 2008 and 2020 revisions.^5,6^ In recent years additional case definitions have been published due to the inability to classify these patients using the EORTC/MSGERC definitions (Table 1).

**Table 1.** Comparison of criteria between the EORTC/MSGERC, *Asp*ICU, IAPA, CAPA and FUNDICU case definitions

	EORTC/MSGERC^6^	(modified) *Asp*ICU^1,7^	IAPA^8^	CAPA^9^	FUNDICU^10^
**Entry criterion**		*Aspergillus*-positive endotracheal aspirate culture	All the following: influenza-like illness positive influenza PCR or antigen temporal relationship	All the following: patient with COVID-19 needing intensive care temporal relationship	
**Host factor**	One of the following: recent history of neutropenia (<0.5 × 10⁹ neutrophils/L for >10 d) temporally related to the onset of invasive fungal disease haematological malignancy receipt of an allogeneic stem-cell transplant receipt of a solid organ transplant prolonged use of corticosteroids at a therapeutic dose of ≥0.3 mg/kg corticosteroids for ≥3 wk in the past 60 d treatment with other recognized T cell immunosuppressants during the past 90 d treatment with recognized B cell immunosuppressants inherited severe immunodeficiency acute graft-versus-host disease grade III or IV, involving the gut, lungs or liver, that is refractory to first-line treatment with steroids	One of the following: neutropenia (absolute neutrophil count <500/mm³) preceding or at the time of ICU admission haematological or oncological malignancy glucocorticoid treatment (prednisone equivalent >20 mg/d) treatment with cytotoxic agents congenital or acquired immunodeficiency			One of the following: influenza COVID-19 moderate/severe COPD decompensated cirrhosis uncontrolled HIV infection with CD4 cell count <200/mm^3^ solid tumours
**Clinical features**	One of the following patterns on CT: dense, well-circumscribed lesions(s) with or without a halo sign air crescent sign cavity wedge-shaped and segmental or lobar consolidation	Abnormal medical imaging by portable chest X-ray or CT scan of the lungs	One of the following: pulmonary infiltrate cavitating infiltrate (not attributed to another cause)	One of the following: pulmonary infiltrate cavitating infiltrate	One of the following: presence of pulmonary infiltrate(s) documented by chest CT presence of cavitation not attributable to other causes
**Signs and symptoms**		One of the following: fever refractory to at least 3 d of appropriate antibiotic therapy recrudescent fever after a period of defervescence of at least 48 h while still on antibiotics and without other apparent cause pleuritic chest pain pleuritic rub dyspnoea haemoptysis worsening respiratory despite appropriate antibiotic therapy and ventilatory support		One of the following: refractory fever chest pain pleural rub haemoptysis	One of the following: fever (38.3°C or higher) persisting after at least 3 d of appropriate antibiotic therapy relapse of fever after a period of defervescence of at least 48 h while still on antibiotics and without other apparent cause pleuritic chest pain dyspnoea haemoptysis worsening respiratory insufficiency in spite of appropriate antibiotic therapy and ventilatory support
**Mycological evidence**	One of the following: microscopical detection of fungal elements or positive culture in BAL, bronchial brush, aspirate or sputum BAL GM ≥1.0 serum or plasma GM ≥1.0 BAL GM ≥0.8 and serum or plasma GM ≥0.7 two or more consecutive positive *Aspergillus* PCR in serum, plasma or whole blood two or more duplicate positive *Aspergillus* PCR in BAL positive *Aspergillus* PCR in serum, plasma or whole blood, and in BAL	One of the following: microscopic detection of fungal elements or positive culture (without bacterial growth) in BAL BAL GM ≥1.0 (modified *Asp*ICU) serum GM ≥0.5 (modified *Asp*ICU)	One of the following: positive culture in BAL (also, aspirate or sputum in case of cavitating infiltrate) BAL GM ≥1.0 serum GM >0.5	One of the following: microscopic detection of fungal elements or positive culture in BAL BAL GM ≥1.0 or LFA ≥1.0 serum GM >0.5 or LFA >0.5 positive *Aspergillus* PCR in serum, plasma or whole blood positive *Aspergillus* PCR in BAL (<36 cycles) positive *Aspergillus* PCR in serum, plasma or whole blood, and in BAL	One of the following: positive culture in BAL BAL GM ≥1.0 serum GM >0.5

*Asp*ICU, clinical algorithm to diagnose invasive pulmonary aspergillosis in critically ill patients; BAL, bronchoalveolar lavage; CAPA, COVID-19–associated pulmonary aspergillosis; EORTC, European Organization for Research and Treatment of Cancer; FUNDICU, fungal infections definitions in intensive care unit; GM, galactomannan; IAPA, influenza-associated pulmonary aspergillosis; LFA, lateral flow assay; MSGERC, Mycoses Study Group Education and Research Consortium.

## Limitations of current IFD case definitions

Alternative IFD case definitions include the *Asp*ICU algorithm.^7^ However, the requirement of having a positive *Aspergillus* culture limited applicability of this algorithm, and up to 57% of patients with severe viral pneumonia could not be classified (Table 2),^1,11–14^ leading to the incorporation of testing for galactomannan (GM) in serum and bronchoalveolar lavage (BAL) fluid.^1^ Case definitions were published for influenza-associated pulmonary aspergillosis (IAPA) and coronavirus disease-19 (COVID-19)–associated pulmonary aspergillosis (CAPA),^8,9^ lacking host factors and relying mainly on positive mycology. More recently, the FUNDICU consensus definition was published to classify critically ill patients with IFD,^10^ but the requirement for chest CT limits the classification of ventilated patients and also mainly relies on mycological evidence (Table 2).

**Table 2.** Ability to classify critically ill patients with structural lung disease, influenza or COVID-19 using current case definitions

	Critically ill patients with structural lung disease^7^ (*n* = 278)	Critically ill patients with influenza^1,11,12^ (*n* = 115)	Critically ill patients with COVID-19^13,14^ (*n* = 81)
EORTC/MSGERC host factor [% (range) patients]	71%	39% (17%–46%)	23% (13%–33%)
Mycological evidence [% (range) patients]			
BAL GM positive (≥1·0)	Not reported	90% (88%–100%)	74% (69%–78%)
Serum GM positive (>0·5)	Not reported	69% (65%–78%)	18% (11%–25%)
BAL *Aspergillus* culture positive	Not reported	67% (63%–89%)	47% (41%–53%)
Classifiable with mycology case definitions [% (range) patients]]			
EORTC/MSGERC^6^	40%^a^	42% (30%–56%)^a^	31% (15%–45%)^a^
*Asp*ICU^7^	100%	61% (58%–89%)^b^	48% (43%–54%)^b^
IAPA^8^	Influenza status not reported	100%	Not applicable
CAPA^9^	Not applicable	Not applicable	100%
FUNDICU^10^	Chest CT data not reported	Chest CT data not reported	Chest CT data not reported

BAL, bronchoalveolar lavage; CAPA, COVID-19–associated pulmonary aspergillosis; EORTC, European Organization for Research and Treatment of Cancer; FUNDICU, fungal infections definitions in intensive care unit; GM, galactomannan; IAPA, influenza-associated pulmonary aspergillosis; MSGERC, Mycoses Study Group Education and Research Consortium.

^a^Unable to classify due to absent EORTC/MSGERC host factor and/or radiological signs; proven cases were irrespective of host factor and radiological signs.

^b^Unable to classify due to absent *Aspergillus* culture (entry criteria); proven cases were irrespective of *Aspergillus* culture.

The increasing number of case definitions is impractical and threatens international standardization and comparisons. Furthermore, IFD definitions need regular updating, with emerging ‘at-risk’ patient groups and the development of new diagnostic strategies.^15^ Previous revisions have taken many years to complete, delaying the inclusion of newly validated diagnostic assays and restricting our ability for timely and enhanced IFD classification. Another limitation is that although a single positive test is considered sufficient mycological evidence to classify a patient with IFD, other discordant (negative) diagnostic tests are not considered. With increasing availability of multiple biomarkers, this criterion likely overstates classification, particularly when other tests, with similar clinical performance, generate discordant results.^16^ Finally, the IFD case definitions primarily support clinical trials and subsequently place an emphasis on specificity when achieving a classification. To increase specificity higher cutoffs are used than recommended by the manufacturers, for example a BAL-GM OD index of 1.0 (Platelia *Aspergillus* Ag assay), whereas 0.5 is recommended by the manufacturer. This limits the applicability of the case definitions for other uses such as clinical diagnosis or IFD surveillance, as the cutoff recommended by the manufacturer is generally used in clinical practice. Given the problems in defining IFD, we need to reconsider how best to classify patients with IFD in routine clinical practice.

## Machine learning–based IFD classification model

**Figure 1.**
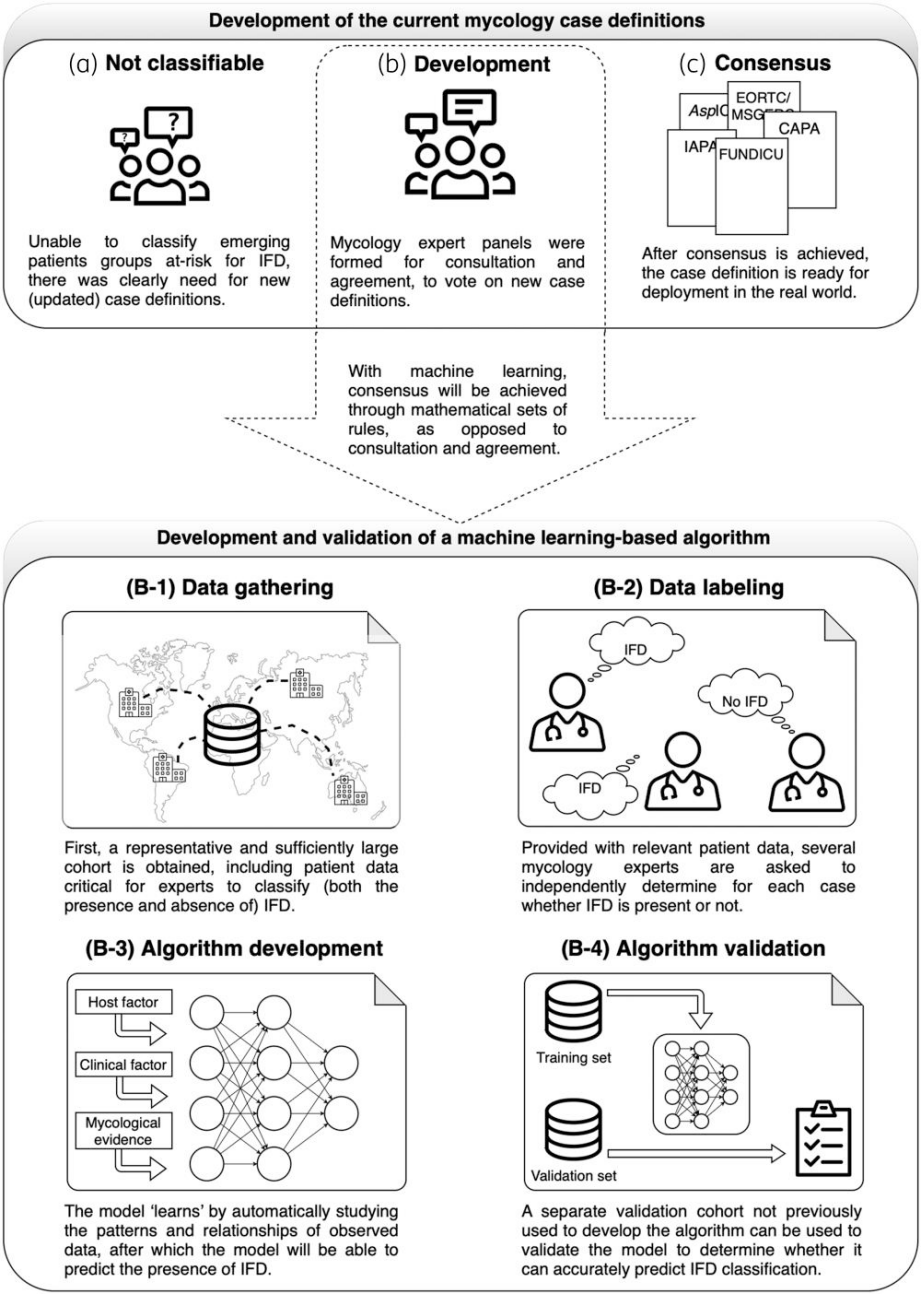
Visualization of development of the current mycology case definitions (upper panel) and the machine learning–based approach (lower panel).

Consensus on the current case definitions was achieved through consultation and discussion between mycology experts, based on relevant literature, knowledge and experience (Figure 1, upper panel). However, given the diverse host–pathogen interactions and the range of diagnostic test combinations/outcomes, it is not feasible to fully integrate this complexity into these manually compiled classifications. Therefore, we investigated if machine learning (ML) could be used to classify patients with IFD. ML models are developed using algorithms—sets of mathematical rules to automatically ‘learn’ from observed data to recognize and describe (complex) patterns and make (more) accurate predictions of future events, saving time and resources while providing consistent outputs. These ML models can continuously learn and improve from new data, allowing them to adapt to changing conditions (e.g. the broadening of host groups at-risk, novel diagnostic tests) and make more accurate predictions over time. ML techniques are increasingly used in infectious diseases, for instance for support in diagnosing bacterial infection in patients with COVID-19,^17^ or to identify risk factors of fungal infections in critically ill patients.^18^

An ML-based approach will help achieve consensus through algorithms (Figure 1, lower panel). First, a representative and sufficiently large cohort is obtained, preferably (multinational) from multiple sites for generalizability, and including patient data critical for experts to manually classify (both the presence and absence of) IFD (Figure 1B-1). When training the algorithm, mycology experts are asked to independently classify the IFD for each case provided (Figure 1B-2), which tells the algorithm what each outcome should be according to the various test combinations and as interpreted by each individual expert. For example, a neutropenic patient positive in serum and BAL by *Mucorales* PCR could be classified with IFD, whereas a critically ill COVID-19 patient positive for BAL *Aspergillus* culture but negative BAL GM might be considered as colonized. The robustness of the ML model can be improved by increasing the number and variety of mycology experts classifying patients with IFD. The model then ‘learns’ by automatically recognizing patterns of observed data (Figure 1B-3), after which the ML model will be able to predict the presence of IFD (e.g. probability) given any combination of host factors, clinical factors and diagnostic test outcomes. Finally, a separate validation cohort not previously used for algorithm development will be used to validate the ML model (Figure 1B-4) to determine whether it can accurately predict IFD classification.

## Towards a unified case definition?

An ML-based IFD classification model may overcome several shortcomings of the current IFD classifications and may be more accurate as it incorporates multiple variables compared with the current definitions. The increased use of electronic patient files and programs to extract specific clinical data increases the feasibility to implement such an approach. However, developing an ML model to classify IFDs in various host groups requires substantial effort. It involves setting up large patient cohorts with harmonized data points and input from multiple mycology experts to classify IFDs for the development and validation of algorithms, achieving consensus through sets of mathematical rules regarding key factors for disease labelling, and subsequent implementation. Nevertheless, given that the second revision of the EORTC/MSGERC definitions took over 12 years to complete, the complexity of the pathology of IFD, and increasing clinical population ‘at-risk’ of IFD, such an endeavour should be considered a worthwhile investment by the mycology community to standardize and reduce the ambiguity in the diagnosis of non-proven IFD.

## Transparency declarations

We declare no competing interests. R.J.M.B. reports grants or contracts from Gilead and Pfizer, consulting fees from Astellas, Basilea, F2G, Gilead and Mundipharma, and payment or honoraria from Gilead and Pfizer, outside the submitted work. A.A. reports grants or contracts from ANR, payment or honoraria from Gilead, and patents from Institut Pasteur, outside the submitted work. K.v.D. reports payment or honoraria from Gilead and Pfizer, and participation on a data safety monitoring board or advisory board for Gilead and Pfizer, outside the submitted work. K.L. reports grants or contracts from TECOmedical, consulting fees from Mundipharma, payment or honoraria from FUJIFILM Wako chemicals Europe GmbH, Gilead, Pfizer and Mundipharma, and support from AstraZeneca, Gilead and Pfizer, outside the submitted work. J.W. reports grants or contracts from Gilead, MSD, Mundipharma and Pfizer, consulting fees from Gilead and Pfizer, payment or honoraria from Gilead, MSD, Mundipharma and Pfizer, support from Gilead, MSD, Mundipharma and Pfizer, and participation on a data safety monitoring board or advisory board for Gilead, outside the submitted work. P.L.W. reports grants or contracts from Bruker and IMMY, consulting fees from F2G and Gilead, payments or honoraria from ACC, Gilead, Mundipharma and Pfizer, and participation on a data safety monitoring board or advisory board for F2G, Gilead and Mundipharma, outside the submitted work. P.E.V. reports grants or contracts from F2G and Gilead, payments or honoraria from F2G, Gilead, Mundipharma and Pfizer, and participation on a data safety monitoring board or advisory board for F2G and Mundipharma, outside the submitted work. All other authors have nothing to declare.
